# Serum Metabonomics Analysis of Liver Failure Treated by Nonbioartificial Liver Support Systems

**DOI:** 10.1155/2018/2586052

**Published:** 2018-07-04

**Authors:** Chunyan Wu, Ying Zhu, Mengqian Yu

**Affiliations:** The First Affiliated Hospital of Dalian Medical University, Dalian 116011, China

## Abstract

**Objective:**

To analyze the small molecular metabolic compounds of nonbioartificial liver for treatment of hepatic failure and make further efforts to study the clinical efficacy, mechanism of action, and pathogenesis of hepatic failure.

**Methods:**

52 patients who met the standard of artificial liver treatment for liver failure were enrolled; these patients included 6 cases of acute liver failure (11.54%), 3 cases of subacute liver failure (5.77%), acute-on-chronic liver failure in 10 cases (19.23%), and 33 cases of chronic liver failure (63.46%). Treatment modes included plasma exchange in 34 patients (65.38%), bilirubin adsorption in 9 patients (17.31%), and hemofiltration in 9 patients (17.31%). The clinical efficacy of artificial liver was assessed by monitoring the effects in the near future. Significant changes in metabolic compounds of liver failure in the treatment before and after artificial liver were screened by using Ultra-Performance Liquid Chromatography-Mass Spectrometry (UPLC-MS). Related metabolic pathways were analyzed by MetaboAnalyst.

**Results:**

After artificial liver treatment, the liver function and coagulation function of liver failure patients were significantly improved (P < 0.01), the Meld score was lower than that before treatment, and the difference was statistically significant (P < 0.05). Serum metabolomics identified 29 small metabolic compounds and 12 metabolic pathways with variable projection importance (VIP) greater than 1 before and after artificial liver treatment. There were 11 metabolic compounds of VIP over 1 and 7 metabolic pathways in the different modes of artificial liver treatment for chronic liver failure. Among them, bile acid metabolism, fatty acid metabolism, and amino acid metabolism are the main sources.

**Conclusion:**

Artificial liver treatment can effectively improve liver function and blood coagulation function and Meld score, clinical symptoms and signs in patients with liver failure; the curative effect of artificial liver was verified, which reflected the clinical value of artificial liver in the treatment of liver failure. Artificial liver treatment of liver failure on fatty acids and primary bile acid synthesis pathway was the most significant. The difference of fatty acid, primary bile acid synthesis pathway, and phenylalanine metabolic pathway in different artificial liver patterns of chronic liver failure was the most significant. This provides a new basis for understanding the mechanism of hepatic failure and the mechanism of liver failure by artificial liver treatment.

## 1. Introduction

Hepatic failure is a serious hepatic damage that can be caused by many factors. In China, the main cause of liver failure is hepatitis B virus (HBV), followed by drugs and hepatotoxicity [1, 2]. Treatment of liver failure includes comprehensive internal medical treatment, artificial liver support therapy, liver transplantation, and other methods. In recent years, the nonbioartificial liver technology has become one of the most effective methods for treating liver failure. It includes a variety of managements, and plasma exchange, hemofiltration, and bilirubin adsorption are the most common ones [3].

Metabolomics is a technique for studying the metabolic network of biological systems. The object which has been researched is mainly the endogenous small molecules with relative molecular mass below one thousand [4]. The most widely used and effective metabolomics techniques are gas chromatography-mass spectrometry [5] and liquid chromatography-mass spectrometry (GC-MS) [6]; the latter is used more often. At present, the use of metabonomics to analyze the changes of serum metabolites in patients with liver failure by nonbioartificial liver treatment is relatively rare. Therefore, changes of serum metabolites before and after nonbioartificial liver treatment were detected by the platform of UPLC-MS, to further analyze the pathogenesis of liver failure and investigate the effect of nonbioartificial liver in patients with liver failure metabolites. This provides a basis for further study of artificial liver technology in the treatment of liver failure. It was expected to provide a new basis for further clinical diagnosis and treatment.

## 2. Materials and Methods

### 2.1. Inclusion and Exclusion Criteria

**Figure 1:**
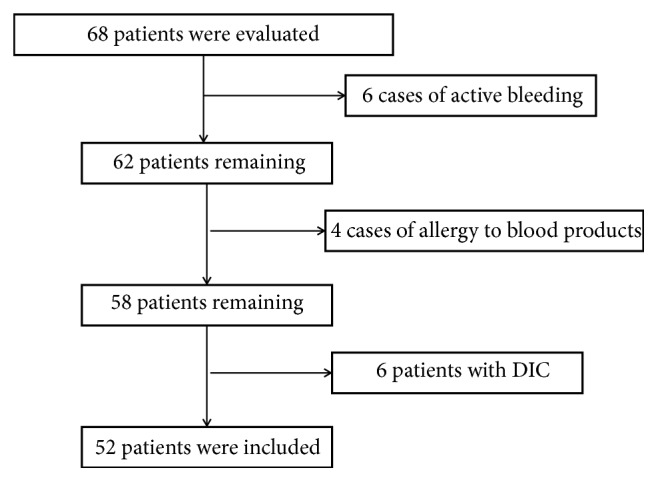
Flowchart of included patients.

All the patients in the group met the criteria for the diagnosis of “guideline for diagnosis and treatment of liver failure” (2012 update) and “guideline for nonbioartificial liver support systems in treatment of liver failure” (2016 update). Liver failure is a serious liver damage caused by a variety of factors and a group of clinical symptoms; the main manifestations include coagulation dysfunction, jaundice, hepatic encephalopathy, and ascites. Acute liver failure is a clinical manifestation of acute onset within 2 weeks. The clinical manifestations of subacute hepatic failure were 2-26 weeks. Acute-on-chronic liver failure is a clinical syndrome with acute or subacute liver decompensation occurring on the basis of chronic liver disease in a short time. Chronic liver failure is a progressive decline and decompensation of liver function on the basis of liver cirrhosis. Exclusion criteria included patients with active bleeding or disseminated intravascular coagulation (DIC); hemodynamic instability in patients; allergic to blood products or medications during treatment. According to the above criteria, 68 patients were evaluated, and 6 cases of active bleeding, 4 cases of allergy to blood products, 6 patients with DIC, and 52 patients with liver failure treated with nonbioartificial liver were selected from December 2015 to November 2016 at the First Affiliated Hospital of Dalian Medical University finally (Figure 1). The aetiology of individual causes of liver failure was different; there were 6 cases of drug hepatitis, 5 cases of alcoholic hepatitis, 3 cases of chronic viral hepatitis B, 2 cases of chronic viral hepatitis E, 3 cases of autoimmune hepatitis, and 33 cases of cirrhosis. According to the diagnosis and classification of hepatic failure, 52 cases were divided into acute hepatic failure in 6 cases (11.54%), subacute hepatic failure in 3 cases (5.77%), acute-on-chronic liver failure in 10 cases (19.23%), and chronic liver failure in 33 cases (63.46%).

### 2.2. Methods of Each Artificial Liver Support

Plasma exchange (PE) is to filter out the plasma containing toxins in the blood out of the membrane and back into the body with the same amount of fresh plasma or fresh frozen plasma with the blood type components that are withheld in the membrane. It can clear liver failure toxins and some pathogenic factors, supplement the essential substances such as coagulation factors that are lacking in liver failure, and correct metabolic disorders caused by liver failure. The disadvantage of PE is that it cannot effectively remove water-soluble substances from small molecules. PE has a certain risk of adverse reactions in the treatment of liver failure, mainly allergic reactions, but symptomatic treatment relieved symptoms and did not affect the treatment effect. Hemofiltration applies a membrane with larger aperture. The pressure difference between the liquid on both sides of the membrane is used as a transmembrane pressure. In the form of convection, the toxins in the blood are removed with water. Hemofiltration is more close to the function of glomerular filtration in the human kidney. If the replacement fluid is contaminated, complications such as fever and septicemia can occur, and this can be avoided by prevention and treatment. The main mechanism of bilirubin adsorption is to separate the plasma from the patient and send the plasma to the bilirubin adsorption column of BS series, so as to completely adsorb the bile acid and bilirubin in the serum and then send the purified plasma back to the human body, so as to effectively remove the blood poisoning metabolites and inflammatory cytokines. Bilirubin adsorption treatment increased the toxin clearance ability, and the safety was high. Studies have shown that PE, hemofiltration, and bilirubin adsorption are safe and effective in the treatment of liver failure and can prolong the survival time of patients.

### 2.3. Defining Criteria to Patients with Different Modalities of Artificial Liver Support

Plasma exchange is the most commonly used method, which can retain the relatively large molecular weight of coagulation factors, hepatocyte growth factor. Hemofiltration is suitable for all kinds of liver failure with acute kidney injury, including hepatorenal syndrome, hepatic encephalopathy, electrolyte imbalance, and acid-base imbalance. Bilirubin adsorption is applied to severe hyperbilirubinemia caused by various reasons and patients with severe cholestasis of liver disease treated by poorly internal medicine. According to the above pattern of artificial liver, 52 patients were divided into plasma exchange in 34 cases (65.38%), 9 cases were bilirubin absorption (17.31%), and 9 cases were hemofiltration (17.31%). Because chronic liver failure accounts for 63.46% of liver failure and plasma replacement is 65.38% of the artificial liver pattern, this article focuses on chronic liver failure with different artificial liver treatment and plasma exchange for liver failure.

### 2.4. Clinical Efficacy Observation Index

According to the guidelines, the clinical efficacy of artificial liver and the changes of the patient's condition were evaluated by monitoring the effects in the near future, including serum bilirubin lowering, PTA or international standardization ratio improvement, MELD score decline, other laboratory indexes improvement, and digestive tract symptom improvement. The metabolic compounds of liver failure were observed before and after artificial liver by using UPLC-MS, and related metabolic pathways were analyzed by MetaboAnalyst.

### 2.5. Instruments and Reagents

The following reagents and instruments were used for this study: 7600 automatic biochemical analyzer from Japan's Hitachi company, blood coagulation analyzer from German BE company, Wasters UPLC-Q Exactive HF MS (Thermo Fisher Scientific, Rockford, IL, USA), centrifuge (Microfuge 22, Beckman Coulter company), vortex mixer (Vortex Genius 3, German IKA group), Acetonitrile and formic acid (from the Fisher company in the United States), ammonium acetate, and analytically pure (from the Sigma-Aldrich company in the United States), and ultrapure water was prepared by the MILLI-Q ultra purified water preparation system (US Millipore Corporation).

### 2.6. Samples Collection and Processing

Fasting venous blood and venous blood after treatment were taken immediately, and the serum was collected after 3500 rpm centrifugation for 8 minutes. Using a constant temperature container with dry ice for transportation, the serum finally were placed in -80 centigrade refrigerator to storage. Serum samples were thawed at room temperature, taking 50 uL serum add into 200 uL acetonitrile containing the internal standard and centrifuging at 10,000 g for 10 min. 180 *μ*l lyophilized supernatant was taken and dissolved in 80 uL volume ratio of acetonitrile/water of 1/4, and, after whirling 30s, centrifugal supernatant was analyzed by LC-MS.

### 2.7. Analysis Condition

Chromatographic condition was as follows: chromatographic column: ACQUITY UPLC HSS T3 (standard: 100 mm × 2.1 mm, 1.8 *μ*m), column temperature: 50°C, and current speed: 0.35 ml/min. Mass spectrometry condition was as follows: MS full scan range of positive ion: 80-1200 m/z; spraying voltage: 3.50 kV; anion: 70-1100; spraying voltage: 3.00 kV. Capillary temperature 300°C, auxiliary gas temperature 350°C. The velocities of sheath gas and auxiliary gas are, respectively, 45 and 10 (arbitrary units), resolution set to 12e^4^.

### 2.8. Data Processing and Statistical Analysis

The raw data collected by mass spectrometry was processed by SIEVE software and 80% principles were used to deal with missing values [7], and data were imported into SIMCA-P 11.5 software, pattern recognition using PLS-DA, and the VIP (Variable Importance in Projection) value of each variable in PLS-DA model was obtained, screening out potential biomarkers. Statistical software was using SPSS 17.0, and P < 0.05 was statistically significant.

## 3. Results

### 3.1. Classification of Liver Failure and Nonbioartificial Liver Patterns

#### 3.1.1. Diagnosis and Classification of Liver Failure

According to the diagnosis and classification of hepatic failure, 52 cases were divided into acute hepatic failure in 6 cases (11.54%), subacute hepatic failure in 3 cases (5.77%), acute-on-chronic liver failure in 10 cases (19.23%), and chronic liver failure in 33 cases (63.46%), Table 1.

#### 3.1.2. Diagnosis and Classification of Plasma Exchange for Liver Failure

Out of 52 patients, 34 cases (65.38%) underwent plasma exchange in the treatment of liver failure diagnosis, according to the classification, divided into 6 cases of acute liver failure (17.65%), 3 cases of subacute liver failure (8.82%), acute-on-chronic liver failure in 10 cases (29.41%), and 15 cases of chronic liver failure (44.12%), Table 2.

#### 3.1.3. Classification of Chronic Hepatic Failure with Different Patterns of Nonbioartificial Liver Treatment

Out of 52 patients, there was chronic liver failure in 33 cases (63.46%); they are in the majority, so the following further on chronic liver failure of artificial liver pattern was analyzed, including 15 cases of plasma exchange (45.454%), 9 cases of bilirubin adsorption (27.273%), and 9 cases of hemofiltration (27.273%), Table 3.

#### 3.1.4. Classification of Nonbioartificial Liver Treatment Patterns

According to the classification of nonbioartificial liver treatment patterns, 52 patients were divided into 34 cases of plasma exchange (65.38%), 17.31% cases were bilirubin absorption, 9 cases were hemofiltration (17.31%), Table 4.

### 3.2. Clinical Efficacy Analysis

#### 3.2.1. Meld Score Analysis of Liver Failure Treated with Nonbioartificial Liver Treatment

The Meld score of all patients before and after nonbioartificial liver treatment was statistically analyzed, and the results showed that the Meld score after nonbioartificial liver treatment was lower than before; the difference was statistically significant (P = 0.001), Table 5.

#### 3.2.2. Clinical Analysis of Liver Failure Treated by Nonbioartificial Liver Treatment

The liver function and coagulation function of the 52 patients treated with nonbioartificial liver were compared and analyzed, and the levels of AST, ALB, ALP, *γ*-GT, TBIL, and PT in nonbioartificial liver were significantly lower than those before nonbioartificial liver treatment (P < 0.01), with statistical significance. The level of ALT after treatment decreased but had no statistically significance (P > 0.05), Table 6.

#### 3.2.3. Clinical Analysis of Plasma Exchange in the Treatment of Liver Failure

When the plasma exchange was used in the treatment of acute liver failure, acute-on-chronic liver failure and chronic liver failure, liver function, and blood coagulation function indicators were compared, and the levels of AST, *γ*-GT, TBIL, PT, and ALB after treatment were significantly lower than before (P < 0.05); the difference was statistically significant, Table 7.

#### 3.2.4. Clinical Analysis of Different Nonbioartificial Liver Treatment Patterns in Patients with Chronic Hepatic Failure

Liver function and blood coagulation function indicators were compared in different patterns of nonbioartificial liver treatment of chronic liver failure, and the levels of AST, *γ*-GT, TBIL, and PT were significantly lower than that before plasma exchange treatment (P < 0.05); there was statistical significance; only TBIL was significantly decreased after bilirubin adsorption treatment (P < 0.05), and there was statistical significant differences; only PT significantly decreased after hemofiltration treatment (P < 0.05), and the difference was statistically significant, Table 8.

### 3.3. Serum Metabonomics Analysis

#### 3.3.1. Serum Metabonomics Analysis of Liver Failure Treated by Nonbioartificial Liver

**Figure 2:**
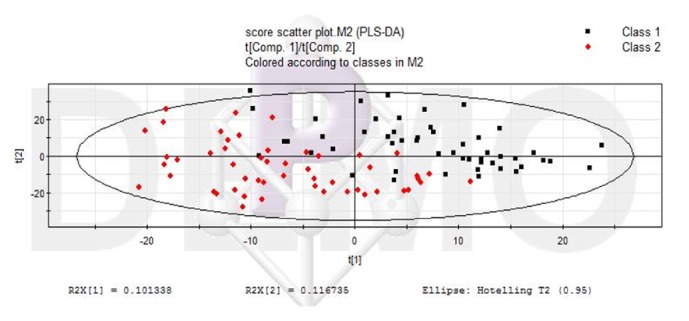
Scoring chart of PLS-DA pattern in treatment of liver failure with nonbioartificial liver.* Note.* Class1, Before nonbioartificial liver treatment; Class2, After nonbioartificial liver treatment.

**Figure 3:**
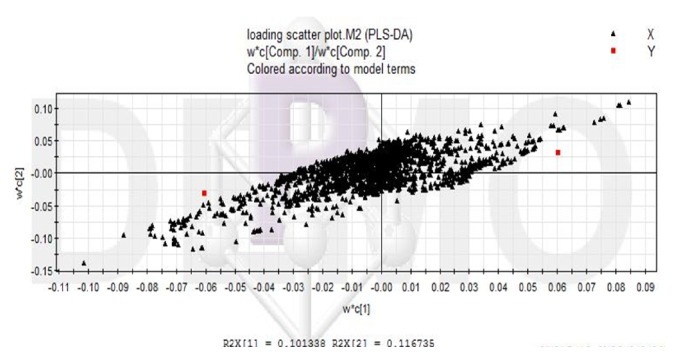
Load chart of PLS-DA pattern in treatment of liver failure with nonbioartificial liver.

**Figure 4:**
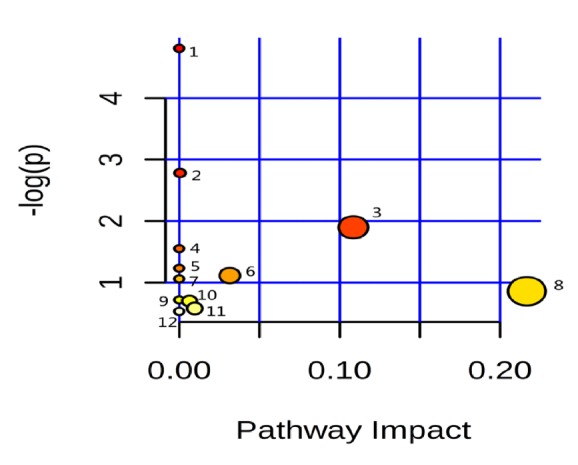
Differential metabolite related pathway. Note that numbers 1-12 represent different metabolic pathways, the name of metabolic pathways, and distribution parameters as shown in Table 10.

The LC-MS data before and after liver failure treated by nonbioartificial liver were processed, and the ion information in negative ion mode was obtained by SIEVE software. All data were processed by PLS-DA analysis and got Figures 2–4. In the scoring chart (Figure 2), the samples were distinguished before and after the nonbioartificial liver treatment, which validated the effectiveness of the nonbioartificial liver treatment; the load graph (Figure 3) showed a number of variables away from the center, indicating that these variables contributed significantly to the model, and there was a significant change before and after the nonbioartificial liver treatment; the validation model showed that R2 = (0, 0.222) and Q2 = (0, -0.228), indicating that the PLS-DA model did not have overfitting, that the prediction between groups was reliable, and that the model was successfully established. We analyzed metabolite with significant difference before and after nonbioartificial liver treatment and VIP greater than 1, which used the MetaboAnalyst analysis. Combined with Figures 2-3, we identified 29 differential metabolites and, as shown in Table 9, the fatty acids and bile acids were taken as the principal thing. Metabolic pathways were analyzed through the MetaboAnalyst analysis, and 12 metabolic pathways were identified, which relates to the proportion of fatty acid metabolites metabolism and bile acid metabolism in the highest (Figure 4, Table 10). These results indicated that nonbioartificial liver treatment had significant effects on fatty acid metabolism and bile acid metabolism.

#### 3.3.2. Serum Metabonomics Analysis of Chronic Liver Failure Treated by Nonbioartificial Liver

**Figure 5:**
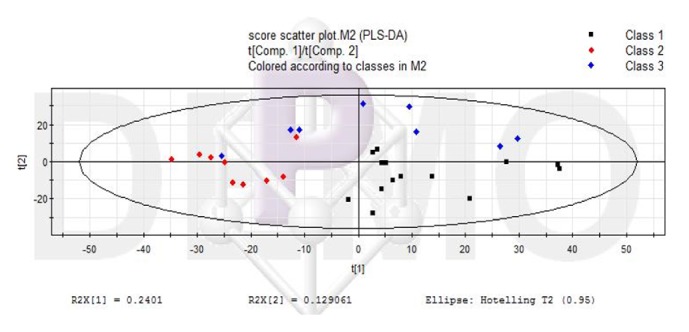
Scoring chart of PLS-DA pattern in treatment of chronic liver failure with nonbioartificial liver. Note the following: Class1: plasma exchange for chronic liver failure; Class2: treatment of chronic liver failure with bilirubin adsorption; Class3: hemofiltration in the treatment of chronic liver failure.

**Figure 6:**
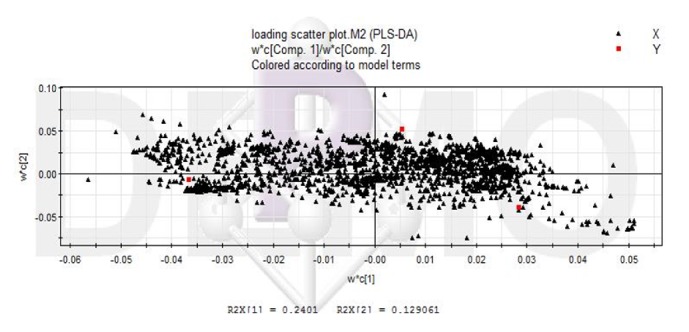
Load chart of PLS-DA pattern in treatment of chronic liver failure with nonbioartificial liver.

**Figure 7:**
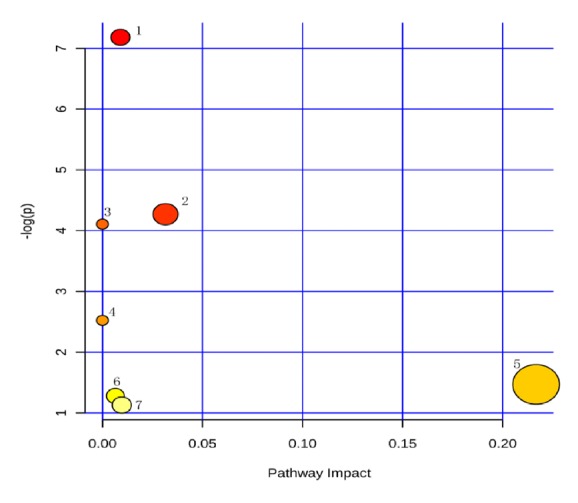
Differential metabolite related pathway in treatment of chronic liver failure with nonbioartificial liver. Note that numbers 1-7 represent different metabolic pathways, the name of metabolic pathways, and distribution parameters as shown in Table 12.

The 52 patients were included, 33 cases of chronic liver failure (63.46%), among them there were 15 cases of plasma exchange (45.454%), 9 cases of bilirubin adsorption (27.273%), 9 cases of hemofiltration(27.273%). Serum LC-MS data of chronic liver failure after treatment with different nonbioartificial liver patterns were analyzed by PLS-DA, and Figures 5–7 was obtained. In the score plot (Figure 5) in different nonbioartificial liver treatment pattern distinguishing, which showed that there were obvious differences between the treatment pattern, load graphs (Figure 6) showed multiple variables away from the center, indicating that these variables are important in distinguishing different treatment modalities; verification model showed that R2 = (0, 0.537) and Q2 = (0, -0.441), which indicated the PLS-DA model without overfitting; internal group prediction model was established successfully reliable. According to the variable importance projection (VIP > 1), combined with Figures 5-6, we identified 11 different metabolites, shown in Table 11, with fatty acids, bile acids, and amino acids; 7 metabolic pathways were identified through the MetaboAnalyst analysis, as shown in Figure 7 and Table 12. The proportion of metabolites involved bile acid metabolism, fatty acid metabolism, and amino acid metabolism in the metabolism was the highest. It indicated that the different treatment patterns had significant effects on bile acid metabolism, fatty acid metabolism, and amino acid metabolism.

## 4. Discussion

Liver failure is one of the most common serious liver disease syndromes, with high fatality rate [1]. There are three main treatment methods for liver failure: medical treatment, nonbioartificial liver support therapy, and liver transplantation treatment [7]. Because of comprehensive internal medical treatment is limited, liver transplantation improved survival rate but affected by the shortage of donor, high cost, and other factors; nonbioartificial liver support therapy can improve liver function, until you find the suitable donor liver or liver regeneration, which is particularly important in the clinical application of liver failure [8–11]. Nonbioartificial liver treatment can significantly improve liver function and coagulation indexes in the early and middle stages of liver failure. It can improve the symptoms in the late stage of liver failure and the survival time of patients waiting for liver transplantation and the survival value of patients [12–14]. In this study, we used metabonomics to analyze the changes of the organism before and after the nonbioartificial liver treatment.

Hepatic uptake of free fatty acids (FFA) plays an important role in the synthesis, storage, and transport of lipids [15]. With necrosis and apoptosis of hepatocytes, the absorbance of free fatty acids decreased and free fatty acids in serum increased liver failure patients. Studies have shown that different forms of fatty acids have different effects [16]. A protective role for endogenously generated unsaturated fatty acids was also indicated by in vivo experiments using genetically modified mice bearing an inactivating mutation in the gene encoding the enzyme stearoyl-CoA desaturase [17]. Exposure of a variety of cell types, including hepatocytes, to long-chain saturated fatty acids led to increased expression of proinflammatory cytokines, inhibition of insulin signaling, induction of endoplasmic reticulum (ER) stress, and promotion of cell death, mainly by apoptosis [18]. After nonbioartificial liver treatment, the levels of various saturated fatty acids decreased and the levels of unsaturated fatty acids increased; this showed that fatty acids were involved in the process of inflammation and repair of hepatocytes. And nonbioartificial liver treatment can effectively affect the distribution of fatty acids in the environment and create conditions for the regeneration of liver cells.

Bile acid is composed of cholesterol in the cytoplasm and microsome of liver cells. Bile acids can be divided into hydrophobic and hydrophilic bile acids according to water solubility [19, 20]. Bile acid enters the cell and can inhibit the oxidative phosphorylation of mitochondria, and the ATP synthesis decreased calcium pump inactivation, extracellular Ca2+ influx, and activation of various proteolytic enzymes, which caused the decomposition of DNA, RNA and protein, cell dysfunction, and ultimately apoptosis [21, 22]. During liver failure, a large number of liver cells were mortified and bile acid was released into the blood, resulting in elevated levels of serum bile acids, and elevated bile acids reaction on liver cells can further lead to necrosis and apoptosis of liver cells. In this study, the hydrophobic bile acid decreased and the hydrophilic bile acid increased after nonbioartificial liver treatment. The positive correlation between hydrophobic bile acids and liver injury was confirmed, and the antagonism between hydrophilic bile acids and hydrophobic bile acids was also confirmed.

The liver plays a key role in amino acid metabolism [23], liver injury can cause imbalance of amino acid metabolism and change of amino acid levels in human body [24, 25]. Phenylalanine is an essential amino acid, it can synthesize proteins and be converted into nonessential amino acid tyrosine. Under the influence of hepatitis virus invasion or other physical and chemical factors, the parenchymal cells of the liver are seriously damaged and even destroyed. Any of various enzymes responsible for biochemical metabolism in the liver cells that are reduced or released into the body fluid for inactivation. Thus, the catabolic pathway of aromatic amino acids slows down, and its content in the blood increases [26, 27]. After the nonbioartificial liver treatment of liver failure, the aromatic amino acids, especially phenylalanine, decreased significantly, indicating the gradual recovery of hepatocytes, which prove that the nonbioartificial liver treatment is helpful for the regeneration of liver cells and the recovery of liver function. In this study, phenylalanine metabolism in patients with chronic liver failure treated with different nonbioartificial liver models were significantly different. It was further proved that the levels of aromatic amino acids changed significantly in the treatment of chronic liver failure by nonbioartificial liver treatment.

In summary, nonbioartificial liver treatment of liver failure mainly through the impact of fatty acid synthesis pathway, primary bile acid synthesis pathway, and phenylalanine metabolic pathway affects the clinical efficacy of patients with liver failure. Through the study of metabolic changes and metabolic pathway of the organism, this study will establish the clinical basis for further study on the pathogenesis of liver failure and the mechanism of nonbioartificial liver treatment.

## Figures and Tables

**Table 1:** Diagnosis and classification of liver failure.

Diagnosis	acute hepatic failure	subacute hepatic failure	acute-on-chronic liver failure	chronic liver failure	Total
Cases	6	3	10	33	52
Proportion	11.54%	5.77%	19.23%	63.46%	100%

**Table 2:** Diagnosis and classification of plasma exchange for liver failure.

Diagnosis	acute hepatic failure	subacute hepatic failure	acute-on-chronic liver failure	Chronic liver failure	Total
Cases	6	3	10	15	34
Proportion	17.65%	8.82%	29.41%	44.12%	100%

**Table 3:** Classification of chronic hepatic failure with different patterns of nonbioartificial liver treatment.

therapeutic methods	plasma exchange	bilirubin adsorption	hemofiltration	Total
Cases	15	9	9	33
Proportion	45.454%	27.273%	27.273%	100%

**Table 4:** Classification of nonbioartificial liver models.

therapeutic method	plasma exchange	bilirubin adsorption	hemofiltration	Total
Cases	34	9	9	52
Proportion	65.38%	17.31%	17.31%	100%

**Table 5:** Meld score analysis of liver failure treated with nonbioartificial liver treatment.

	Before treatment	After treatment	The value of P
Meld score	23.38 ± 5.32	21.38 ± 4.81	0.001*∗*

*Note.* The Meld score before and after nonbioartificial liver treatment was P ⩽ 0.001, which was statistically significant.

**Table 6:** Clinical analysis of hepatic failure treated by nonbioartificial liver treatment.

Index	Mean (before and after)	Standard deviation (front and back)	The value of P
ALT(U/L)	117.06, 69.60	236.50, 76.28	0.051
AST(U/L)	185.15, 141.23	250.61, 138.12	0.003*∗*
ALB(g/L)	30.59, 28.33	4.61, 2.81	0.001*∗∗*
ALP(U/L)	95.54, 86.33	33.33, 31.24	0.01*∗*
r-GT(U/L)	60.77, 49.06	36.66, 21.87	0.003*∗*
TBil(umol/L)	372.53, 263.53	229.01, 170.05	0.001*∗∗*
PT(s)	24.66, 19.74	8.36, 5.82	0.001*∗∗*

*Note.∗*Compared with the preclinical indexes in the nonbioartificial liver treatment P < 0.01. *∗∗*Compared with the preclinical indexes in the nonbioartificial liver treatment P ⩽ 0.001. There was significant statistical significance.

**Table 7:** Clinical analysis of plasma exchange in the treatment of liver failure.

Liver failure type	Index	Mean (before and after)	Standard deviation (front and back)	The value of P
acute hepatic failure	ALT(U/L)	130.00, 76.33	147.77, 51.69	0.08
	AST(U/L)	180.50, 100.17	117.18, 52.11	0.028*∗*
	ALB(g/L)	31.82, 25.98	3.26, 1.06	0.042*∗*
	ALP(U/L)	124.33, 93.00	24.19, 21.93	0.027*∗*
	r-GT(U/L)	139.83, 79.83	30.65, 18.14	0.028*∗*
	TBil(umol/L)	383.88, 206.82	127.49, 71.11	0.028*∗*
	PT(s)	19.12, 16.87	2.50, 1.03	0.046*∗*

subacute hepatic failure	ALT(U/L)	427.67, 313.67	20.79, 104.18	0.285
	AST(U/L)	343.67, 367.33	24.66, 267.99	1.00
	ALB(g/L)	34.83, 28.00	2.60, 7.43	0.109
	ALP(U/L)	117.00, 67.67	22.61, 21.50	0.109
	r-GT(U/L)	56.33, 31.67	12.50, 13.58	0.109
	TBil(umol/L)	633.90, 384.87	67.76, 80.85	0.109
	PT(s)	37.77, 20.17	4.80, 1.80	0.109

acute-on-chronic liver failure	ALT(U/L)	272.90, 88.00	469.06, 56.50	0.008*∗∗*
	AST(U/L)	371.20, 187.80	506.46, 193.74	0.005*∗∗*
	ALB(g/L)	33.24, 29.85	1.92, 1.74	0.007*∗∗*
	ALP(U/L)	74.00, 66.00	36.24, 25.79	0.541
	r-GT(U/L)	70.50, 54.80	20.70, 13.10	0.047*∗*
	TBil(umol/L)	559.37, 408.53	380.74, 321.44	0.005*∗∗*
	PT(s)	20.24, 15.37	6.26, 1.53	0.028*∗*

Chronic liver failure	ALT(U/L)	37.8, 37.53	27.83, 18.69	0.955
	AST(U/L)	97.20, 78.20	90.85, 76.31	0.019*∗*
	ALB(g/L)	28.60, 27.86	6.21, 1.96	0.394
	ALP(U/L)	89.00, 80.13	28.71, 23.39	0.053
	r-GT(U/L)	41.40, 36.40	19.73, 18.53	0.033*∗*
	TBil(umol/L)	319.73, 223.11	145.33, 79.61	0.001*∗∗∗*
	PT(s)	24.06, 17.86	5.78, 2.60	0.001*∗∗∗*

*Note.∗*Compared with the preclinical indexes in the treatment of plasma exchange P < 0.05. *∗∗*Compared with the preclinical indexes in the treatment of plasma exchange P < 0.01. *∗∗∗*Compared with the preclinical indexes in the treatment of plasma exchange P ⩽ 0.001. There was significant statistical significance.

**Table 8:** Clinical analysis of different nonbioartificial liver treatment patterns in patients with chronic liver failure.

Non-bioartificial liver treatment method	Index	Mean (before and after)	Standard deviation (front and back)	The value of P
Plasma exchange	ALT(U/L)	37.80, 37.53	27.83, 18.69	0.955
	AST(U/L)	97.20, 78.20	90.85, 76.31	0.019*∗*
	ALB(g/L)	28.60, 27.86	6.21, 1.96	0.394
	ALP(U/L)	89.00, 80.13	28.71, 23.39	0.053
	r-GT(U/L)	41.40, 36.40	19.73, 18.53	0.033*∗*
	TBil(umol/L)	319.73, 223.11	145.33, 79.61	0.001*∗∗*
	PT(s)	24.06, 17.86	5.78, 2.60	0.001*∗∗*

Bilirubin adsorption	ALT(U/L)	55.33, 60.22	40.57, 38.87	0.05
	AST(U/L)	162.56, 178.33	70.30, 79.48	0.123
	ALB(g/L)	29.14, 28.82	4.32, 3.76	0.859
	ALP(U/L)	106.00, 104.78	20.71, 23.65	0.398
	r-GT(U/L)	54.00, 58.67	12.51, 13.56	0.095
	TBil(umol/L)	301.80, 210.37	90.99, 33.98	0.011*∗*
	PT(s)	20.89, 21.38	5.76, 2.66	0.213

Hemofiltration	ALT(U/L)	25.56, 26.11	12.33, 6.95	0.767
	AST(U/L)	97.89, 109.44	85.78, 99.71	0.260
	ALB(g/L)	30.21, 28.61	3.22, 1.90	0.066
	ALP(U/L)	93.56, 102.56	40.73, 45.90	0.097
	r-GT(U/L)	37.78, 39.44	21.38, 21.15	0.233
	TBil(umol/L)	229.00, 220.31	106.43, 92.20	0.441
	PT(s)	33.64, 27.87	9.01, 8.88	0.011*∗*

Note: *∗*Compared with the preclinical indexes in the treatment of nonbioartificial liver in Chronic liver failure P < 0.05, *∗∗*Compared with the preclinical indexes in the treatment of nonbioartificial liver in Chronic liver failure P < 0.001. There was significant statistical significance.

**Table 9:** Identification of characteristic metabolites.

Number	Detection of mass/charge ratio	The theory of mass/charge ratio	metabolite	VIP value
1	112.1	113.1	creatinine	2.96
2	391.3	392.6	Ursodeoxycholic acid	2.74
3	367.2	288.4	Dehydroepiandrosterone	2.70
4	203.1	204.2	L- tryptophan	2.12
5	217.0	200.1	1,3 hydroxy uric acid	2.07
6	178.1	178.2	benzoyl-glycine	2.05
7	281.3	282.5	FFA 18_1	1.84
8	204.1	205.2	indolelactic acid	1.81
9	303.2	304.5	FFA 20_4	1.60
10	448.3	467.6	Glycerol deoxycholate	1.52
11	309.3	310.0	FFA 20_1	1.46
12	185.2	186.3	FFA 11_0	1.39
13	407.3	408.6	cholalic acid	1.37
14	167.0	168.1	uric acid	1.37
15	391.3	392.6	deoxycholic acid	1.35
16	157.1	158.3	FFA 9_0	1.33
17	339.3	338.6	FFA 22_0	1.32
18	286.3	284.5	FFA 18_0-d3	1.32
19	305.3	306.3	FFA 20_3	1.30
20	199.2	200.3	FFA 12_0	1.29
21	241.2	242.4	FFA 15_0	1.28
22	391.3	391.6	Ursodeoxycholic acid	1.28
23	171.1	172.3	FFA 10_0	1.25
24	295.3	296.3	FFA 19_1	1.24
25	337.3	338.4	FFA 22_1	1.23
26	301.2	302.5	FFA 20_5	1.20
27	369.2	269.4	DHES-3	1.20
28	528.3	449.4	glycoursodeoxycholic acid	1.19
29	498.3	499.7	Tauroursodeoxycholic Acid	1.18

*Note.* FFA: free fatty acid; DHES-3: 3-dehydro estrone; VIP: variable importance in projection.

**Table 10:** Metabolic pathway name and distribution parameter.

Number	Metabolic pathway name	Ordinate value (-log(p))	Abscissa value (pathway impact)
1	Fatty acid synthesis	4.8098	0.0
2	Primary bile acid synthesis	2.7816	5.4E-4
3	Tryptophan metabolism	1.8992	0.10853
4	Phenylalanine, tyrosine and tryptophan biosynthesis	1.5525	0.0
5	Nitrogen metabolism	1.233	0.0
6	Phenylalanine metabolism	1.1137	0.0315
7	Glycine, serine and threonine metabolism	1.061	0.0
8	arachidonic acid metabolism	0.85981	0.21669
9	Aminoacyl-tRNA biosynthesis	0.71939	0.0
10	Arginine and proline metabolism	0.70068	0.00645
11	Purine metabolism	0.57911	0.00969
12	Steroid hormone biosynthesis	0.53171	0.0

*Note. *The number of 1-12 corresponding to the 1-12 metabolic pathway in Figure 4.

**Table 11:** Identification of characteristic metabolites about chronic liver failure with nonbioartificial liver.

Comparison of treatment methods	Detection of mass/charge ratio	The theory of mass/charge ratio	metabolite	VIP value
Plasma exchange and Bilirubin adsorption	167.0	168.1	uric acid	2.03
	407.3	408.6	Cholic acid	1.61
	112.1	113.1	creatinine	1.58
	391.3	392.6	anthropodesoxycholic acid	1.16
	303.2	304.5	FFA 20_4	1.09
	167.0	515.7	cholaic acid	1.11
	286.3	284.5	FFA 18_0-d3	1.08

Plasma exchange and Hemofiltration	167.0	168.1	uric acid	2.03
	407.3	408.6	cholalic acid	1.61
	112.1	113.1	creatinine	1.58
	391.3	392.6	anthropodesoxycholic acid	1.16
	303.2	304.5	FFA 20_4	1.09
	281.3	282.5	FFA 18_1	1.40
	178.1	178.2	hippuric acid	1.33
	203.1	204.2	L- tryptophan	1.16

Hemofiltration and Bilirubin adsorption	281.3	282.5	FFA 18_1	1.40
	498.3	281.2	phenylacetylglutamine	1.28

*Note.* VIP: variable importance in projection.

**Table 12:** Metabolic pathway name and distribution parameter.

Number	Metabolic pathway name	Ordinate value (-log(p))	Abscissa value (pathway impact)
1	Primary bile acid synthesis	7.1808	0.009
2	Phenylalanine metabolism	4.2699	0.0315
3	Fatty acid synthesis	4.1066	0.0
4	Taurine and hypotaurine metabolism	2.5233	0.0
5	arachidonic acid metabolism	1.4694	0.21669
6	Arginine and proline metabolism	1.2801	0.00645
7	Purine metabolism	1.1293	0.00969

*Note.* The number of 1-7 corresponding to the 1-7 metabolic pathway in Figure 7.

## Data Availability

The data used to support the findings of this study are available from the corresponding author upon request.

## References

[1] (2012). Chinese Medicine Association Infectious Diseases Branch Liver Failure and Artificial Liver. *Chinese Journal of Hepatology*.

[2] McPhail M. J. W., Kriese S., Heneghan M. A. (2015). Current management of acute liver failure. *Current Opinion in Gastroenterology*.

[3] (2016). Chinese Medical Association of Infectious Diseases Branch of liver failure and artificial liver group, Treatment of Hepatic Failure with Abiotic Artificial Liver. *Chinese Journal of Clinical Infectious Diseases*.

[4] Hai T. L., Lian L. (2007). The successful practice of metabolomics in medical research. *Progress in Modern Biomedicine*.

[5] Krone N., Hughes B. A., Lavery G. G., Stewart P. M., Arlt W., Shackleton C. H. L. (2010). Gas chromatography/mass spectrometry (GC/MS) remains a pre-eminent discovery tool in clinical steroid investigations even in the era of fast liquid chromatography tandem mass spectrometry (LC/MS/MS). *The Journal of Steroid Biochemistry and Molecular Biology*.

[6] Drexler D. M., Reily M. D., Shipkova P. A. (2011). Advances in mass spectrometry applied to pharmaceutical metabolomics. *Analytical and Bioanalytical Chemistry*.

[7] Zhen M., Yun W. (2016). Treatment status of liver failureif possible. *Journal of Clinical Hepatology*.

[8] Sarin S. K., Kedarisetty C. K., Abbas Z. (2014). Acute-on-chronic liver failure: consensus recommendations of the Asian Pacific Association for the Study of the Liver (APASL) 2014. *Hepatology International*.

[9] Lee J., Lee D., Lee S. (2017). Functional Evaluation of a Bioartificial Liver Support System Using Immobilized Hepatocyte Spheroids in a Porcine Model of Acute Liver Failure. *Scientific Reports*.

[10] Chen J., Huang J., Yang Q. (2016). Plasma exchange-centered artificial liver support system in hepatitis B virus-related acute-on-chronic liver failure: a nationwide prospective multicenter study in China. *Hepatobiliary & Pancreatic Diseases International*.

[11] Schilsky M. L. (2011). Acute liver failure and liver assist devices. *Transplantation Proceedings*.

[12] Li M., Sun J., Li J. (2016). Clinical observation on the treatment of acute liver failure by combined non-biological artificial liver. *Experimental and Therapeutic Medicine*.

[13] Nyberg S. L. (2012). Bridging the gap: Advances in artificial liver support. *Liver Transplantation*.

[14] Xu X., Liu X., Ling Q. (2013). Artificial Liver Support System Combined with Liver Transplantation in the Treatment of Patients with Acute-on-Chronic Liver Failure. *PLoS ONE*.

[15] Musso G., Gambino R., Cassader M. (2009). Recent insights into hepatic lipid metabolism in non-alcoholic fatty liver disease (NAFLD). *Progress in Lipid Research*.

[16] Mantzaris M. D., Tsianos E. V., Galaris D. (2011). Interruption of triacylglycerol synthesis in the endoplasmic reticulum is the initiating event for saturated fatty acid-induced lipotoxicity in liver cells. *FEBS Journal*.

[17] Li Z. Z., Berk M., McIntyre T. M., Feldstein A. E. (2009). Hepatic lipid partitioning and liver damage in nonalco-holic fatty liver disease: role of stearoyl-CoA desaturase. *Biological Chemistry*.

[18] Akazawa Y., Cazanave S., Mott J. L. (2010). Palmitoleate attenuates palmitate-induced Bim and PUMA up-regulation and hepatocyte lipoapoptosis. *Journal of Hepatology*.

[19] Poupon R. E., Chrétien Y., Poupon R., Paumgartner G. (1993). Serum bile acids in primary biliary cirrhosis: Effect of ursodeoxycholic acid therapy. *Hepatology*.

[20] Sagawa H. (2001). Protection against hydrophobic bile salt-induced cell membrance damage by liposomes and hydrophilic bile salts. *American Journal of Physiology*.

[21] Rolo A. P., Palmeira C. M., Wallace K. B. (2003). Mitochondrially mediated synergistic cell killing by bile acids. *Biochimica et Biophysica Acta (BBA) - Molecular Basis of Disease*.

[22] Nakajima T., Okuda Y., Chisaki K. (2000). Bile acids increase intracellular Ca. *British Journal of Pharmacology*.

[23] Kinny-Köster B., Bartels M., Becker S. (2016). Plasma amino acid concentrations predict mortality in patients with end-stage liver disease. *PLoS ONE*.

[24] Holecek M. (2015). Ammonia and amino acid profiles in liver cirrhosis: Effects of variables leading to hepatic encephalopathy. *Nutrition Journal *.

[25] Plauth M., Schütz T. (2011). Branched-chain amino acids in liver disease: New aspects of long known phenomena. *Current Opinion in Clinical Nutrition & Metabolic Care*.

[26] Jiangping W. (2003). *Determination of Aromatic Amino Acids in Serum by High Performance Liquid Chromatography and Its Clinical Application*.

[27] Kong M., Hongbo S., Bai L., Yao J., Chen Y. (2016). Changes and Characteristics of Plasma Amino Acid Spectra in Patients with Hepatitis B Virus Related Chronic Hepatitis, Clinical meta. *Cirrhosis and Chronic Hepatic Failure*.

